# Qualitative description of outreach and engagement in perinatal substance treatment in Finland

**DOI:** 10.1186/s13011-022-00513-y

**Published:** 2023-01-21

**Authors:** Minna Sorsa, Maria Hohenthal, Miia Pikulinsky, Hanna Sellergren, Kaija Puura

**Affiliations:** 1 Child Psychiatry, Tampere University Hospital, Pirkanmaa Hospital District, Tampere, Finland; 2 Nursing Science, Faculty of Social Sciences, Tampere University, Biokatu 12, FM 5 (4-306), SF-33520 Tampere, Finland; 3 The Federation of Mother and Child Homes and Shelters, Helsinki, Finland; 4 Faculty of Medicine and Health Technology, TamCAM Research Centre, Tampere University, Tampere, Finland

**Keywords:** Early intervention, Engagement, Family, Low threshold, Outreach, Perinatal period, Substance use, Women

## Abstract

**Background:**

Women with perinatal substance problems experience a multitude of barriers to care. They have specific early intervention needs, they endure societal stigma, and both substances and mental health issues influence the way they navigate within support and treatment systems. Early interventions for women with perinatal substance problems are underresearched contexts. The aim of the study is to describe building relationships and engagement within an outreach and low threshold service encounter tailored for pregnant women with SUD (substance use disorder).

**Methods:**

The data consist of online written narratives from 11 workers involved in the program and feedback from 504 families in the recovery process comprising 228 open-ended answers. The data were analyzed with a thematic analysis.

**Results:**

The programs are characterized by flexibility and the implementation of inclusive ways to approach families. The themes for enhancing relationships and engagement within outreach and low threshold programs are *Acceptance and attitude: a sensitive approach of approval*; *flexibility within strictness to allow for diversity and individuality*; *availability and space to ensure a trustful atmosphere*; *negotiating* via *doing to build connections*; and *everyday life changes: imagining recovery*. The themes represent the need of being available, focusing on the worker’s attitudes and building connections by doing together, and visioning recovery together.

**Conclusions:**

The study results can add to the understanding of SUD outreach and low threshold work during pregnancy. The elements described in this study need further theoretical development, research and critical assessment. Building relationships during pregnancy were characterized by connecting within everyday life situations and supporting the development of an attachment relationship between the baby and the parents. To promote recovery, a comprehensive approach in which substance-related issues and mental health conditions are interconnected can be favored. Engaging early on during pregnancy might enhance success during future rehabilitation.

## Introduction

In recent years and during the COVID-19 pandemic, substance problems of pregnant women have become more severe and involve more psychiatric conditions, difficulties, and challenges in everyday life management [1]. At the same time the treatment options may be understaffed or lacking, with access to care for co-occurring disorders remaining limited [2]. Unfortunately, it seems that current interventions do not reach the most vulnerable families with SUD, e.g. those with small children, or parents suffer from mental ill health or come from minority groups [2–5]. This article focuses on the outreach and engagement of women in interdisciplinary substance use treatment, as early interventions for women with perinatal substance problems are underresearched contexts [6, 7]. Research on co-occurring and complex disorders recommends approaches to create involvement in treatment using specific clinical attitudes and welcoming ways to meet with clients [8]. Within the systems of care for pregnant women, enhanced support, endorsing positive aspects of treatment, a focus on gender-relevant topics, and the use of motivational enhancement have been associated with improved attendance among pregnant women with SUDs [5, 6, 9, 10]. Pregnancy and motherhood are motivating forces to increase interest in help-seeking and making changes. They can, however, also function as barriers against disclosing mental health and substance use problems as mothers may be afraid of children being taken into custody [2, 5]. Therefore, women with multiple vulnerabilities will need extra support in accessing treatment. The latest gender-sensitive service development contains integrated and coordinated approaches, where substance treatment is coordinated with Child Protection Service (CPS) programs, childcare, mental health treatment, mother-infant attachment and interaction. Services have had good experiences with relational-based approaches, particularly when the women enroll during pregnancy or immediately after childbirth [2]. Strengthening the personal skills, resources and self-efficacy of the mothers can help to improve the health of both the mother and the newborn [10], while punitive policies and practices discourage access to SUD treatment during pregnancy [2, 5, 10, 11]. Stigma in society towards substance-using people is pervasive, as policies and practices blaming people for their problems exist, undermining both scientific evidence and the personal resources and motivation of pregnant women [10, 11]. Societal disapproval of women’s use of substances [7, 12, 13] or failing the societal expectation of being a “good mother” can cause women to feel shame, regret, and guilt [2, 9, 14–17], and make them feel that they do not have a voice. Women perceive more barriers to treatment than men [12, 18], such as lack of childcare [2, 6], and they are less likely than men to enter treatment [2, 12, 19–24]. There is also a scarcity of tailored treatment for women during pregnancy, as most SUD treatment focus on men. Men outnumber women in substance treatment [6, 22], as women with alcohol disorders tend to underutilize treatment. Only 8.7% of pregnant women receive specialized substance treatment, even though buprenorphine and methadone treatments prevent relapse and treatment attrition [6]. The gender-based stigma may not recognize the probability of women suffering physical or sexual abuse, gender-based violence, trauma histories, social exclusion, or social role problems [7, 16, 25]. It may be particularly difficult for women with problematic substance use to leave an abusive relationship because of contextual and complicating dynamics within relationships to protect the partner or children [16, 17]. Economic constraints, the need for shelter, and the fear of retaliation challenge the option of leaving a violent partner, even in situations of extreme emotional abuse, and many women choose to maintain secrecy even in front of available and helpful resources [16].

Barriers to treatment may follow from choices within the health and social care system delivery level, as affordable and available services for substance use in pregnancy may be scarce in many areas, such as in the US [10, 11]. We will study an outreach and low threshold specialized substance treatment within primary care in Finland, where the well-baby clinics are available for free for every pregnant woman and the family members. In this context the services were arranged by the local municipalities on the basis of national legislation until 1.1.2023 [26–28], when the new well-being areas were established in the new social welfare and health reform. Family support policies in Finland encourage individualized interpretations of family problems, where adults learn to use their own capabilities [29]. The policy aims to detect and screen the needs of children and their parents as early as possible, and the family may be guided to special support services. The primary screen for substance use is the AUDIT, and for perinatal depression, it is the Edinburgh Postnatal Depression Scale (EPDS) [30]. Early intervention universal parenting support focuses on strengthening the parent–child relationship and improving parenting skills [29, 31]; Child protection services (CPS) and social work for families also support families in challenging life situations and may utilize home service, family work, support persons, support families, and other primary care services. Well-baby clinics provide counseling and support for child development and prevention of mental ill health in families. All pregnant families have free access to professional support for parenting, building healthy home environments, and promoting healthy habits. The coverage of well-baby clinics is 99.7% of pregnancies [30]. Currently, a national network of Huumeet, alkoholi, lääkkeet (HAL)-outpatient clinics for women focusing on drugs, alcohol, and medication are located in connection with maternity outpatient clinics or gynecological outpatient clinics in several areas of Finland [1, 30]. In 2017, 94% of the referrals were from well-baby clinics [30]. Between 2016 and 2020, approximately 1000 women visited this specialized service yearly [1]. Drug use is illegal in Finland, and opioid substitution treatment was approved in Finland in the late 1990s, with the first needle exchange program opening in 1997 [32]. There is no compulsory treatment during perinatal time due to substance use. Yearly, approximately 600 families in Finland attend specialized substance rehabilitation programs for pregnant families with substance issues. In 2021, there were 13 rehabilitation units, where 40% of the clients were women, 40% were children and 20% were fathers or partners [1]. These specialized programs can be considered women-centred, which is a difference to the general client profiles within SUD treatment. The National Holding Tight Treatment System (HTTS), administered by the Federation of Mother and Child Homes and Shelters, provides a special level of social service for pregnant mothers or for fathers with their children under three years and their siblings.

There is also a gap in the research literature in regard to help-seeking during the perinatal period among women with problematic substance use and co-occurring psychiatric disorders [6]. Specific comprehensive services have been developed that simultaneously tackle several problem areas and focus on relationship building, reciprocity, and involving whole families in treatment [9]. However, these principles are not yet known in treatment initiations within a comprehensive and continuous framework. Early first-line interventions are necessary in society to promote family mental health [33], as parental mental health and child well-being are interconnected. Solutions for the issues of mental ill health and problematic substance use have been extensively sought within preventive mental health care by earlier recognition of illness [34]. Screening is used widely, even though screening during pregnancy within SUD has not been recommended as a universal tool, as it may in itself be stigmatizing [10].

Treatment of SUD during pregnancy is challenging, because mothers with SUD often have histories of trauma from their early interpersonal childhood experiences, resulting in trauma as a precursor of substance use and impacting parenthood and child development [5, 35, 36]. Trauma exposure affects an individual’s capacities for emotional understanding and processing. Trauma-informed integrated treatment contains the co-occurring substance and mental health issues. Positive results in incorporating a trauma-informed approach arise from emphasizing treatment engagement and building relationships via therapeutic alliances [9, 37]. The trauma-informed approach is especially important at the start of help-seeking and in engaging with the services, since early dropout is frequent, and past insecurities and parental uncertainty need to be expressed within caring and nonjudgmental clinical environments [36, 38]. Relational continuity of care has been shown to be important, especially for women suffering from depression [39]. Relational models of parenting interventions involve the recognition of attachment and building relationships because the internal working models of women contain complex mental representations of the self, the caregiver, and the quality of relationships [36]. Women may perceive and interpret their distress as a personal failure, and they may fear disapproval from their social network and therefore do not seek help. If a mother is insecurely attached herself, she may have negative preconceptions of others, experience high levels of avoiding attachment, have low levels of trust in others, and be less willing to seek professional help [40].

There is a need to develop accessible, gender-sensitive services and approaches that challenge the barriers and stigma, and even coercion women are facing when seeking help in the perinatal period [2, 7, 10, 11, 19, 22, 41]. A personalized approach that promotes a feeling of being valued may enhance active engagement [42]. Engagement can mean commitment to using services [42, 43], or it can be defined as a process where an inner experienced level of engagement emerges [44]. This study will focus on describing and analyzing a specialized outreach and low threshold service practice for women. This approach could add to the current understanding of low threshold services, assertive outreach programs, the promotion of gender-sensitive and trauma-informed services. Accessing safe spaces and building trust are important, and intertwined with engagement, as these are associated with greater service use [38]. Our study within the outreach and low threshold services for pregnant women with SUD aims to describe building relationships and engagement within the outreach and low threshold service encounter tailored for pregnant women.

### Methods

#### Data collection

The data collection occurred at the Holding Tight Treatment System (HTTS) administered by The Federation of Mother and Child Homes and Shelters. The first Mother and Child Home for women with problematic substance use was founded in 1990, and the national HTTS was founded in 1998. Staff has since been trained in community care, reflective functioning (mentalizing), early interventions and intergenerational trauma [15]. The theoretical perspectives are attachment, trauma and mentalization. HTTS has seven Mother and Child Homes specializing in substance treatment and eight open ward units. Rehabilitation includes supporting parenting and early interaction and special baby-oriented substance rehabilitation for pregnancy and the first three years. It is especially important to provide treatment as early as possible during pregnancy. The goal is to secure the healthy growth of the infant and to motivate the parent into intensive rehabilitation. Rehabilitation for lasting changes requires sufficient time and planning between the families and multidisciplinary professionals. The service is focused on women-centred care, as in a profile of the service users in 2015–2018, most clients were women (62%), and most were in the age group of 25–29 years (29%) (Table 1). In 2015–2018 in HTTS, the rehabilitation phases lasted most often over two months (Table 2).

**Table 1:** Age and gender distribution of clients in HTTS recovery programs during 2015–2018

Clients	Women (*n* = 448, 62%)	Men (*n* = 271, 38%)	Total (*n* = 719, 100%)	%
Age 24 or under	130	30	160	22
Age 25–29	155	51	206	29
Age 30–34	101	71	172	24
Age 35 or over	62	119	181	25

**Table 2:** Number of families and length of stay in recovery programs during 2015–2018

Length of stay	Number of families (n)	%
Under 6 days	7	1
1 to 2 weeks	17	3
3 to 4 weeks	26	5
5 to 8 weeks	45	9
2 to 6 months	146	30
7 to 12 months	125	25
12 to 18 months	64	13
18 to 24 months	34	7
Over 24 months	29	6
Total	493	100

HTTS started to implement and develop a low threshold outreach program (ETMA) in 2018, which aims to increase the accessibility of services and reduce stigma. The program utilizes the same theoretical framework as HTTS; it supports abstinence and promotes parenting and early interaction. Families consist of the mother, baby, siblings of the baby, and a partner, whether living in the same home or elsewhere. ETMA is active in social media work; they run peer support groups and chat discussions online, aiming at early support. The work contains harm reduction, such as promoting discussion about the substances and stigma related to it. They also collaborate with CPSs, well-baby clinics, and different low threshold sites, where families can join anonymously. The program participation can be anonymous and take place flexibly at several locations. In 2021, most participants in the low threshold program were women (67%) and in the 18- to 29-year age group (Table 3). There are no similar statistics available on outreach work, including social media, chat discussions or support groups.

**Table 3:** Age and gender of clients in ETMA low threshold service in 2021

Age	0–2 years	7–17 years	18–29 years	30–62 years	Total	%
Female	3	1	55	32	91	67
Male	4	1	12	28	45	33
Total	7	2	67	60	136	100

Of all visits in 2021, the pregnant woman participated in 42% of the meetings, 20% were nonpregnant women, and 29% were partners/fathers. The most common substance in 2021 was alcohol (27%), and illicit drugs (17%), mixed usage of substances or medications (38%), and the substance was not known (18%).

The data consist of staff and client data. All staff members (*n* = 14) within ETMA were approached in September 2018 with an e-mail from within the organization, which led the staff members to an online query. The eleven participants all came from a tailored ETMA program specifically addressing the needs of women with problem substance use and small children. Additionally, data contain feedback collected in HTTS from 504 families in 2015–2018, after the families with small children had ended their rehabilitation phase (Table 1 and Table 2 describe the families).

The eleven female workers come from six different units in different locations in Finland. Their ages were 28–52 years, with an average of 43 years. They had worked at HTTS for 4–25 years, on average 13 years. Three of the workers had nursing, and eight workers had social work training backgrounds. The staff participated via online written narratives. They were asked to describe their outreach and low threshold work, what knowledge and skills they need and the specificities of the ETMA program. They were asked to reply via open-ended questions:

1. Describe your low threshold work (what you do, how you do, where you do),
2. Describe your outreach work (what you do, how you do, where you do),
3. What knowledge and skills do you need when working within the low threshold?,
4. What knowledge and skills do you need when working within outreach?,
5. What does this work require from you as a human and as a worker?,
6. What is relevant in regard to work success?,
7. What is difficult in low threshold work, tell an example?,
8. What is difficult in outreach work, tell an example?,
9. What is special in the low threshold work of ETMA model?,
10. What is special in the outreach work of ETMA model?, and
11. Whether there was something else to add.

The responses and data from staff consist of approximately 7000 words.

Researching the area of treatment initiation and engagement within SUD services is very difficult because women and families may not recall what happens when help-seeking occurs, as many arrive while experiencing traumatic life events. The choice of this study is to ask clients their feedback afterwards in regard to the early phases and the relationship with workers. Many clients arrive with referrals from well-baby clinics or CPSs and may be strongly recommended to participate. All women may not feel they attend voluntarily. Therefore, an appropriate timing for interviewing the women is after the recovery phases have been completed. HTTS families give both numerical and open-ended feedback when their recovery phase ends. Workers connected with the parents and wrote down the oral feedback. Families were asked:

1. Open feed-back about the rehabilitation phase, and
2. To express development needs.

Altogether, 504 persons (ca 70%) out of the 719 persons in total had given feedback in 2015–2018. Families gave feed-back in the end of rehabilitation (*n* = 306), half year after the recovery phase (*n* = 124), and one year afterwards (*n* = 74). The respondents were mothers (*n* = 204), fathers (n = 74) or the respondent was not known (*n* = 226). Feedback was given during four years: 2015 (*n* = 97), 2016 (*n* = 95), 2017 (*n* = 180) and 2018 (*n* = 132). All feedback is included as data for the study. The family feedback from HTTS was available as paper prints, and all texts consisting of approximately 8000 words were digitized to enable verbal analysis with software. The feedback contained 228 open answers related to early help-seeking and encounters with staff, which forms the basis of the qualitative data set of client and family feed-back.

#### Data analysis

The special interdisciplinary perspective of the ETMA system might be of interest to other service providers and function as an asset for developing general services. We are placing the substance use of women in a cultural and practical context, deriving from previous theoretical frameworks. The selected services have a unique contribution to the research question [45]. The qualitative data are analyzed with a thematic analysis, which is especially well suited for an underresearched area [46]. The data analysis process was as follows: 1) thorough reading; 2) sentences responding to the research question were inductively coded from both data sets using QSR NVivo to facilitate the process; 3) the themes were described; 4) the two data sets were combined; and 5) the themes were contested in discussions within the study group.

Rigor in the study was established via several tools [47]. We have described how we obtained the two data sets and completed the data analysis. We wanted the themes to be descriptive and as authentic as possible so that the report could be useful and utilized further, e.g., for theory development. The themes reached depth and contained contrasting viewpoints. The background section of the article describes the standpoint from which we have approached the data. The validity questions regarding thematic analysis relate to whether the data are an accurate reflection of the whole data set [46]. Therefore, all themes include excerpts from the original data. Confirmation occurs in relation to previous research, and the original excerpts assist in highlighting new perspectives [45] within engagement in SUD treatment for women and during pregnancy. We have also contextualized the study findings within a specific national context. Additionally, we incorporated the COREQ evaluation of the study process [48].

#### Ethical questions of the study

The ethical aspects of the study were reviewed and approved by the management of the Federation of Mother and Child Homes and Shelters. Staff gave their consent to participate prior to participation. The mothers and families responded voluntarily to a regular feedback by the time they ended their rehabilitation. No names of sites are utilized during data analysis or reporting the findings to protect the respondents. The families’ feedback was anonymous by start. The data from staff were collected with an online tool administered by HTTS, and after this, only the 1st author (MS) worked with the data to ensure confidentiality and anonymity. The data from mothers and families were collected by HTTS anonymously with the register files containing solely year and site of feedback.

### Results

**Fig. 1:**
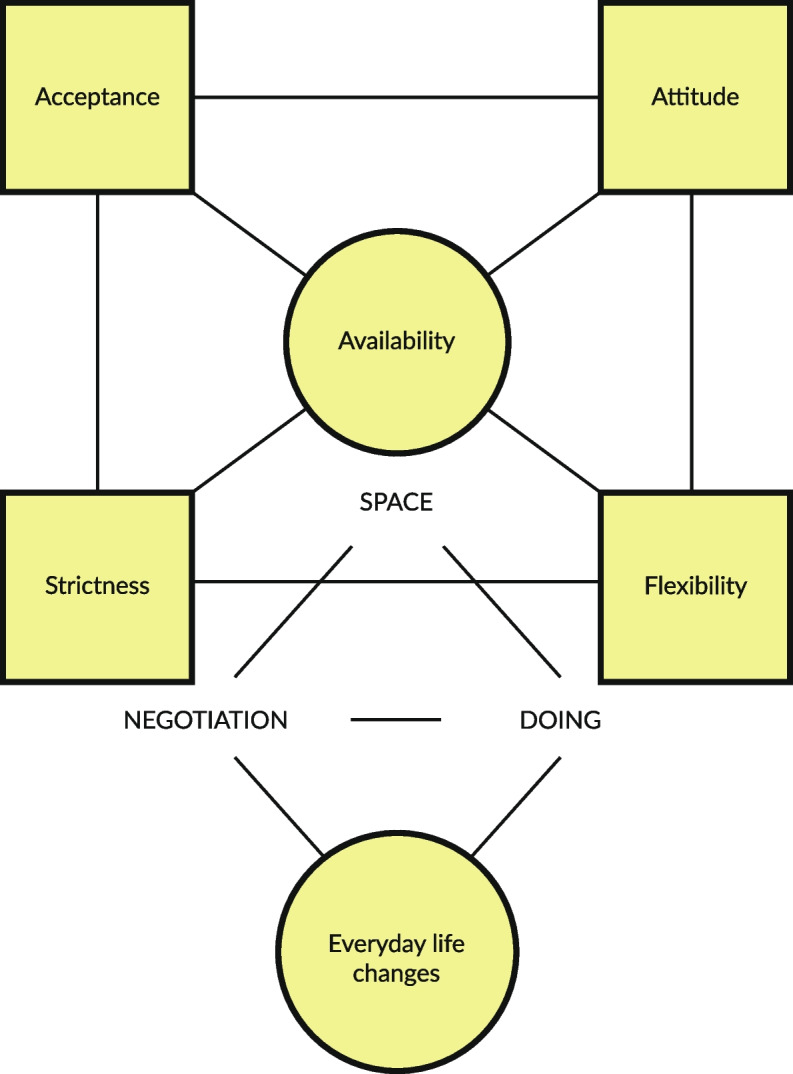
Building relationships and engagement within outreach and low threshold services

This study will focus on describing and analyzing specialized outreach and low threshold program practices for women with problematic substance use. The research question is as follows: *How are relationships and engagement formed within an outreach and low threshold service for pregnant women with SUD?* The themes for enhancing relationships and engagement within outreach and the low threshold program will then be described (see Fig. 1).

The women’s backgrounds are very diverse when they connect with the services; since the women are at a different stage of pregnancy, they may be first-time mothers or already have older children. They use different substances, drugs, or alcohol or misuse medications. Some suffer from psychological distress or depression, and others endure chronic physical illnesses. Women may be active users of substances when meeting with them the first time. Not everybody is motivated to participate in the ETMA program.

When attending the program, the clients carry with them their memories and the stigma they experienced during encounters with previous service providers or within society. The health care professionals’ attitudes at childbirth may be memorized and feel painful, despite inner knowledge of their right to equal access to supportive services and their right to proper treatment (*hyvä kohtelu*), “*It’s about human values*,” not undermining or being arrogant about the women’s requests for help:

*“Wishes a general change of attitudes in regard to substance abuse. Especially when a woman is pregnant, doctors and nursing staff in obstetrics and primary care may treat those with substance-use problems really badly. Experts-by-experience type of connections work the best.” (Client, 126).*

If previous requests for help have been negative, many women prefer not to disclose in the future. It may be surprising for the woman to find out that people and staff at the ETMA program can be friendly, and in fact, kindness may in itself function positively in regard to the women’s previous experiences within the substance world involving threatening events and experiences.

#### Acceptance and attitude: a sensitive approach of approval

The staff has a specific attitude when connecting with women experiencing this special life situation with problematic substance use and motherhood. Staff members have in-depth knowledge about substances within the contexts of addiction, illness, and lifestyle that are threatened to be challenged when women seek help. Because their backgrounds are versatile and their life situations are diverse, meeting with professionals entails individualization, flexibility, and adjustments to the staff’s perspectives and the working models employed. Staff members within ETMA need skills to assess the help-seeker within a larger societal and cultural frame or phenomenon — the very many diverse levels and forms of substances have an impact on women arriving at the services. The personal agency of a woman is contested when she seeks help for her pregnancy or with motherhood.

According to families and staff, the best results occur if staff can begin collaborating with families during pregnancy. Staff aim to create an atmosphere of acceptance to contrast the stigma the women may have experienced prior to ETMA. The women must feel as if they are welcomed; the saying “*come as you are*” is applicable in the overall theme of acceptance, and a tailored approach is favored. Furthermore, a sensitive approach of approval can create connection:

*“A suitable environment and workers..important to understand the human irrespective of their starting point.” (Client, 442).*

The first encounter is described as especially important from the staff perspective. In the beginning, the mother, child, and healthcare professional need opportunities to become closer to each other. Trust can be built in an open atmosphere, and it benefits the woman and the staff in the event that a relapse occurs later, so that additional challenges can more easily be disclosed. Women want to be supported and encouraged in the process. They need positive feedback. The workers can have a positive impact by demonstrating that they believe in the mothers:

*“It has been important that somebody believes in me, even though I do not always believe in myself. Even though we only chat a little bit, I might later feel that the discussion was useful.” (Client, 210).*

Many staff members were perceived as warm. Women noted that after their rehabilitation phase, they had also received help within other areas that they had not thought of in advance. In situations when they feel tired, this fatigue may restrict their options to ask for and receive help. From a woman’s perspective, help-seeking requires honesty and openness, and emotions arising during staff encounters may feel good, bad, irritating, or even anxiety-provoking. When the women felt physically ill, they sometimes felt they had to repeatedly repeat themselves and explain their situation to be taken seriously:

*“As a tip to other clients, openness is the most important. You cannot be helped if you do not share anything about yourself.” (Client, 384).*

The clients admitted that their own attitudes could impact their help-seeking efforts. Moreover, the timing of their access could impact their ability to adhere to treatment or how they interpreted staff attitudes. Treatment also felt like an ambiguous period because they were living and learning about their emotions.

The clients noted the importance of not blaming them. The clients considered that it was most important to find their motivation themselves and to pursue progress even though it did not always feel useful. Many women come from well-baby clinics after a positive substance use screening or if they shared information on their close family’s substance use. The staff perspective is decidedly oriented toward the well-being of the fetus and developing “*baby in the mind*” thinking:

*“Working throughout the pregnancy to ensure the growth and development of the fetus, so the baby would be born healthy, despite exposure. Fathers, if known, and if they want to be involved in the family, should have access to rehabilitation at the same time as the mother.” (Worker).*

Staff considers the baby, pregnancy, and the effects related to expectancy every time they are able to meet with the woman or soon-to-be mother. Staff try to answer any questions the mothers have, and they discuss how the baby is doing. The pregnancy period gives a woman the chance to focus on her own life because self-centeredness will be more difficult after the child has been born. She might have difficult experiences in her life that have not yet been addressed and require attention. It may take a long time until motivation emerges. During the phase when a connection is being formed with staff, many women may experience difficulty accepting care. To make a trusting relationship work, staff need their own personality while still maintaining a professional distance.

#### Flexibility within strictness to allow for diversity and individuality

Women may feel afraid or ambivalent by the time they arrive at the service encounter, partly because of the risk of their children being taken into care unless their substance abuse issues are resolved:

*“Child protection feels scary, but it is important they are strict by start…staff wants to support, provide feedback that it is going well, and that keeping their child is possible.” (Client, 476).*

The openness in communication includes explaining why urine tests are taken and using screens with the aim of assessing for possible continuing substance use. Staff also discuss the child’s wellbeing and the impact of substances on the child’s development.

When working with these women, enhancing the development of trust and attempting to enhance the collaborative relationship between staff and clients is continuous. From the staff perspective, supporting and encouraging the parenting agency of women in situations while also setting necessary boundaries requires a combination of flexibility and strictness. Flexibility helps when a woman forgets to tell staff that she cannot make it to her scheduled meeting, cancellations occur, and meeting hours or the number of meetings are changed. The situation can be reviewed and re-evaluated even quickly, such as in the event of physical health conditions. The reason for this flexibility stems from the raison d’être of HTTS:

*“To hold tight to the clients and try to make them engage in some form of support… if a mother cancels a meeting, I try to reach out to her and clarify, why the meeting did not occur; if I cannot reach her, I’ll go and visit the mother at her house if necessary. I only make home visits to clients I already know. On the other hand, the need to approve, if the mother does not want to accept support.” (Worker).*

Flexibility and alertness to the possibility of making changes is needed in the event of a relapse. If a collaborative relationship has commenced, a relapse may mean that the woman will need additional support for a while. More testing and screening are occasionally administered. The respondents believed that an individualized flexible approach was possible if a worker was more knowledgeable about the special family questions. Respecting the woman’s choice and agency means that it remains the mother’s right to decide how to set boundaries in the event of challenges. During these times, it is not possible to strictly follow plans, and flexibility may be needed:

*“It is important to go through failures to have an experience that overall, it is possible to move forward, and failures do not ruin it all.” (Client, 369).*

The process also involves disappointment if a relapse causes a client to stop attending her meetings or a relationship cannot be formed with a specific client. For workers, understanding that a relationship cannot be formed with every client may be a frustrating emotional journey to process. Workers know that noninterrupted intensive help has been shown to be the most effective. Beginning collaboration is a long-term effort, and it is important to also hold tight during difficult moments. Clients assert that individuality gives them the opportunity to recover. As humane people, staff challenge the stereotypes of addicts; the individuality that is necessary to connect with the mothers involves respect and is woman-centred. The clients emphasize the individuality of each event and of each woman and mother in relation to their own personal goals, as these need to be the women’s goals, not the workers’.

Because trust was developed in person-to-person connections, a contrast can be seen in whether it is possible for the women to speak out in groups. This may not be possible due to sensitive questions and issues related to the culture residing in communities within addictions, such as interpersonal violence in the drug world. If a close person such as the baby’s father continues using substances, it will be challenging for the mother to continue toward her goal of recovery.

A family-centered approach supports the individualized perspective. Under Finnish law, workers must report living conditions that would challenge or harm the child’s well-being to social care. On the other hand, work at HTTS is baby-centred. Therefore, staff utilize an honest, direct, and friendly approach to cope with such situations while developing a relationship. While there is a risk that the woman will leave and not commit to treatment in the outreach and low threshold phase, many clients giving feedback were in favor of coercion:

*“Perhaps when I hid things and in a certain way you knew, you could have faced me with the truth and asked, ‘What the shit are you doing?’” (Client, 185981).*

Because other clients were against coercion and felt that the focus on the baby was oppressive, many questions required negotiations between the clients. Staff needed to confirm the mother, since the aim at ETMA is to make the mother understand the viewpoint of her child. In the beginning in the outreach and low threshold program, there is more flexibility and less confrontation than in future recovery steps. According to clients and workers, strict boundaries do not work in outreach. The challenge is how individualized boundaries are perceived by peers, who may insist upon the exact same rules for everyone in the rehabilitation communes. Because speaking openly is important, workers choose the point of confrontation with sensitivity, so as not to interfere with the development of trust.

*“You should be able to talk openly about things without sanctions or fear. Speaking honestly is very important. It is difficult to be honest—for example, about stealing or other problems—because of fear of children being taken into custody.” (Client, 362).*

Insensitive confrontations may lead the individual to choose to be silent or be offended if trust has not yet been developed.

#### Availability and space to ensure a trustful atmosphere

Low threshold staff are available in easily accessible locations. Outreach services can meet women anywhere, and when some level of connection has been established, home-based low threshold care can also become an option. Being available to the woman’s needs utilizes calmness within a specific space:

*“Meeting, hearing the other human, respect, calmness in the situation—you must not hurry the other—and verbalizing and bringing up difficult issues with respect.” (Worker).*

The women may feel vulnerable (*rikki*) if they have not yet attended preventive care. Their diverse background and experiences impact the manner in which workers can connect with them. Workers and clients emphasize the need for easily accessible care:

*“It is important that it would be easy to seek care and to be given the chance and understanding of the enabling change involved in motherhood.” (Client, 134).*

Availability is a space that contains a sense of freedom because the person seeking help is fully entitled to speak within a trustful atmosphere. Again, staff utilize sensitivity to create the feeling of non-pressure and availability. Since each human has a unique background, they also require unique solutions. A woman’s specific situation may necessitate changes in their personal manner of connecting with other humans within the services or when creating trust. Developing trust, starting to discuss her own problems, and asking questions about her life situation emerge in stepwise co-creative processes. Staffing changes may interrupt building a connection and may lead to setbacks. Situations where the child’s well-being requires action may not be pleasant experiences for the woman.

Many families positively mentioned home-based outreach, their everyday lives, and rhythm with children as possibilities for learning mothering and fathering in the context of their own home. The service can develop into a trusted safe space that women can contact by choice. Clients said they could trust the ETMA-built trust, which ensured that someone would always connect with them. Home-based support in their everyday lives was favored as easy access to care for families with children or when a client had a bad day. The goal of recovery and everyday situations could function as a frame for discussion and availability.

#### Negotiating via doing to build connections

The experiences of being heard and receiving positive feedback can strengthen a woman’s ability to function. Because meetings between humans occur in specific times and spaces, several negotiations are needed regarding different common rules of collaboration. After connection between the woman and workers has been co-created, a question may arise as to who is responsible for the recovery. The woman’s agency may grow when she makes decisions and has the freedom of choice, such as what to do within the spaces where connections occur:

*“A family-cafe would be great, so that parents could meet freely, exchange views, discuss bothering matters and children could play. There could be workers, if something acute and worrying would occur and the workers role would mainly be an enabling bystander, she should no way intervene, unless the clients want.” (Client, 225).*

Clients said that meeting with peers was considered an ideal empowering option that would allow for the exchange of views from the inside and would shift the workers from an advisory role to consulting when they were needed. While some women required more encouragement when participating in peer groups, other clients refused participation because not everyone has the energy to be involved. If a woman was sensitive to criticism, workers negotiated with them with the aim of respectful counseling or feedback:

*“Sometimes, the workers had too much advice and hints in motherhood and less would have been sufficient, considering that a new mother may be sensitive to too much counselling. I missed receiving more positive feedback.” (Client, 168584).*

Negotiations and interactions between clients and workers are intertwined with details and impressions and involve taking directions when communicating during different shared actions. Many interactions in the outreach and low threshold programs occur by doing: sitting together to have coffee, visiting different places such as parks, going for walks or engaging in sports activities, relaxing, going out on day excursions, engaging in the arts or photography work, baking or beginning a new hobby, and finding additional recreation activities. Supporting motherhood by engaging in activities may further enhance the creation of a connection and participating in the program. Because verbal expression may be difficult for some women, doing provides more opportunities for building connections.

Doing together and commencing activities may lead to the development of interest in living everyday life and in devising solutions such as learning how to manage money. Furthermore, the positive emotional component of engagement yields new experiences. It was suggested that the women need to connect with others, have fun together, and find everyday activities that give them a sense of meaningfulness. These activities can be seen as empowering and help them enjoy their everyday life. One of the clients suggested that connecting with peers might be helped by participating in activities:

*“These would be easier at the start, to be with others and meet them.” (Client, 137).*

Aiming to establish a rhythm in their everyday lives also benefitted by doing together with a worker. Concrete everyday skills and seeking solutions could be elaborated by using a pregnancy diary:

*“In every meeting, we go through the mother’s pregnancy diary together, and we reflect on how the baby is doing and what the mother should know and remember in relation to the pregnancy. The mother has a task to ponder during every week of the pregnancy. I use cards and assignments.” (Worker).*

While there were suggestions that the whole family would be included in treatment, other formats were also suggested: women-only peer support groups, groups for single mothers, father groups, and groups for both parents if they are together. Many felt that the relationship between the mother and father was also an issue that needed attention.

#### Everyday life changes: imagining the recovery

In early help-seeking efforts, clients connect with visions of what they perceive can become their future. During the first steps, it becomes necessary to view the future and discover potential goals. For recovery to occur, connections within ETMA and HTTS are co-created. This process creates the option of stronger personal agency and yields insights into a woman’s everyday life:

*“Accepting oneself the way you are, searching for your own strengths.” (Client, 501).*

The workers stated that women must be able to define their goals themselves so that goals from outside the individual do not become overwhelming and impossible to implement at a practical level. A woman needs to elaborate upon her substance problems, her everyday life, and parenting at her own pace. Engagement and involvement may take a long time. Several clients expressed a need to hear the long recovery perspective and the possible phases of full recovery from the start, at the stage after the client connected with professionals:

*“I would have wished more information about the whole process from start. Everything came so quickly that it was sometimes difficult to relate to things. E.g. information on how long the rehabilitation takes and what things help it to end. In the beginning it would be good to go through all details of rehabilitation. In the end, rehabilitation has felt really good, and I have learnt much about myself, and I have strengthened myself, even though it felt difficult at start.” (Client, 483).*

Increased knowledge about recovery phases may enhance the vision of a future experience of being helped. Committing to and engaging with the forthcoming recovery process requires a certain level of preparedness for the necessary steps thereof:

*“Rehabilitation should be long enough—at least a year—because changes do not occur quickly.” (Client, 338).*

From the client viewpoint, the first steps involve being informed about their rights and the manner in which the overall helping system functions. The women need to know that self-directed reflections are part of the forthcoming recovery process. Women needed information about details such as the importance of reviewing their life histories as a whole, digging into problems with self-esteem, and how to maintain their individual point of view.

Timing is of interest, since coming for a visit to ETMA and increasing interest in recovery from problematic substance use during pregnancy is an opportunity for the mother to focus on her own care. The earlier a woman shifts the focus to herself and the baby about to be born, “*the baby in the mind*” knowledge promotes the family’s future steps. After the baby is born, learning to cope with the everyday routines in the family’s life will require significant energy. As such, the focus on the child involves understanding the child and becoming prepared to live with an infant. In the next step, children are involved in the recovery steps of the mother and her partner. New skills can be learned in everyday situations:

*“The child at the center: children accompanying them in rehabilitation. The children’s joy and being together without drugs was rewarding and important.” (Client, 98).*

The women received concrete guidance in childcare and knowledge about child growth and development. Rehabilitation focuses on the interaction between the child and the parent in the context of addictions, everyday skills, presence and engagement. The skills emerge stepwise. The knowledge of hardships ahead and perseverance is therefore helpful at the start. Workers considered it important that the parents’ own skills would awaken so they could see their own resources:

*“The individual meetings usually include a discussion on the mother’s situation and how she is doing, and for every meeting, I have a theme related to the baby/motherhood/parenting planned.” (Worker).*

An orientation to the future also exists in the knowledge that any future rehabilitation could be temporary, and the main aim for the women and their families would be to eventually live their everyday lives without using the service. If a bond has already formed between the mother and baby during pregnancy, the family can commit earlier to recovery-oriented communes and will start to hear about eg schedules and can imagine their way forward. They will learn basic skills according to their needs, such as nurturing and eating habits within the family, maintaining a daily rhythm, and basic interactive and parenting skills.

A question about an orientation to the future emerged from the client responses, which suggested the need for increased visibility and the presence of the program within other services and in well-baby clinics:

*“More visibility perhaps, survival stories, etc… Communicating more.” (Client, 259).*

Clients use Google to create a pre-understanding and learn about early interventions, “points of entry,” and the different options available to them prior to accessing these services. In the current Finnish context, families can learn about suitable and available services, and they use peer recommendations.

Workers felt that awareness of HTTS and ETMA work with families in the early years was scarce. Other workers within health and social care fields may not know the client group, which may cause misunderstandings, misconceptions, and even stigma. As such, the workers networked among professionals that serve the same clientèle, and some provided open groups in well-baby clinics. They regularly meet with the network and release information in the form of leaflets and as digital materials, and they are also aware of online content strategies and a presence on the Internet on social media platforms such as Twitter, Instagram and Facebook, where social media is accessible to the professional network and to clients. Staff marketed their work; in this context, outreach utilizes networking and virtual channels.

## Discussion

The results of our study characterize professional practices in relation to perinatal substance use and demonstrate how outreach and low threshold services are administered in gender-sensitive, trauma-informed and non-coercive ways. Because current interventions do not reach the most vulnerable families with small children [2–5], it is necessary to develop programs within outreach and low threshold services for women and families during the perinatal period. The barriers earlier described regarding social stigma, coercion and punitive practices, socially restrictive relationships, partner substance use, and feelings of shame and guilt [2, 6, 7, 9–12, 14, 15, 19, 21–25, 37, 40, 41, 49, 50] can be counteracted with the comprehensive, integrated solutions of the HTTS and ETMA programs. It is noteworthy, that even in the presence of early interventionist systems of care, most women do not seek help or they have problems identifying the most relevant ways to access care [1, 41].

In contrast to punitive and coercive practices [10, 11], our data were collected at a service focusing on inclusion and engagement from the theoretical perspectives of intergenerational trauma, attachment, early interaction and mentalization [9, 15]. The themes covered in our study capture how outreach and low threshold interventions benefit from the approach. For example, the theme *“Acceptance and attitude: a sensitive approach of approval”* strongly emphasizes building relationships, which stand at the core of attachment-based and trauma-informed interventions [9, 15, 35, 40, 51, 52]. The need for a welcoming approach identified in relation to comorbidity involving substance use and mental health conditions [8] was also addressed in our study. According to our results, skilled staff need to demonstrate greater sensitivity in approaching women with problematic substance use or SUD—for instance, need to avoid confrontation—and instead cultivate trust. The women in our study also benefited from a gender-sensitive approach [2, 7, 19, 22, 41], which helped to create connections and engagement [42, 44].

The theme of *“Flexibility within strictness to allow for diversity and individuality”* captured how the work had to be focused on children’s well-being, while the gender-sensitive approach emphasized the women’s agency to make their own decisions in any situation regarding their own lives. In that process, staff needed to be strict when CPSs needed information, and sometimes extra screens had to be administered. In general, CPSs are responsible for ensuring that each child’s right to a safe, stimulating environment for growth is protected. If an outreach or low threshold program emphasizes individuality and diversity within a family-focused frame, it may be easier to find solutions in which women feel approved and accepted as themselves, not as *“addicts”*. In Finland in general, society and the community take responsibility for substance use problems [17, 32]; however, our data also clarify that the women themselves need to start becoming active in their lives in order for recovery to proceed [29]. In turn, that undertaking can allow them to identify their strengths and develop agency.

The ETMA program, intended for all members of the family, focuses on the mother as the first step to potentially increasing self-awareness [24] because the period of pregnancy may afford women time to reflect on their lives and possible sources of trauma. Such self-reflection can help women develop skills in reflective functioning [9, 15, 51] before the birth of their children because after childbirth, they are required to devote attention to their babies and nurture relationships with them. The practice used in outreach and low threshold services contains a strong future orientation and targets changes in everyday life and a commitment to recovery. Along those lines, the theme *“Everyday life changes: Imagining the recovery”* included the recognition of some possible future steps. As captured by the theme *“Availability and space to ensure a trustful atmosphere”*, the ETMA program took place within the community, as well as virtually with the aim of creating safe spaces where it would be appropriate to address challenging questions stemming from their everyday life. This is why the theme *“Negotiating via doing to build connections”* is relevant, as change is based on everyday life activities.

The biggest difference between the outreach and low threshold services and rehabilitation were the acceptance of the slowness of improvements and the ability to endure uncertainty until a connection with the women had been created. After a collaborative relationship had been established, it made it possible for the workers to explain their worries concerning the clients and the treatment process to clients in greater detail. To overcome stigma, staff members need to recognize that help-seeking may be challenging for clients given their background and that developing trust with them will take time. Both workers and clients indicated the ideal of equality. Whereas staff expressed that every human shares the same reality irrespective of their life situation, clients wished that professionals would take a gentler approach. To imagine their recovery, clients suggested making more information about the whole process available online and appreciated the possibility of investigating the process before committing to treatment or seeking help. Promoting the well-being of such clients will require raising public awareness of the existing ETMA and HTTS services and marketing them. By informing and messaging about substance treatment, for example, on social media and other platforms, knowledge about such services will reach those in need. Providing the general public with information about services and SUD treatment may mitigate the stigma related to substance treatment. The possibility of attending anonymously is important, and first-line services should be free of cost.

The societal context in which women use substances during pregnancy imposes certain requirements and gender roles, including being a good mother, and judgements of the women’s character can emerge among health care professionals as well [2, 7, 10, 53]. Professionals who blame women for using substances during pregnancy may not recognize the women’s suffering due to, for example, physical and/or sexual abuse, gender-based violence, and/or trauma, all of which have often been associated with substance use [5, 7, 16, 17, 52]. The flexibility and availability of services, including the possibility of at-home visits where women can feel safe and be candid, can be developed in parallel. Beyond that, connections could be built outside official relationships with staff while engaging in a wide range of activities, particularly by using resources of one’s own choosing and interacting with peers. To build connections, many negotiations were required in such activities, which corroborates past findings showing individuals’ willingness to participate [12, 49] and well-being in everyday life despite challenges [41, 54]. Women need nonjudgmental access to care, and they may benefit from promoting involvement in their communities as well [40, 41]. It is noteworthy that the complex vulnerabilities women may experience should not cause clients to be treated as deficient or lacking [10, 53]. Many problems may be longstanding and unlikely to be quickly resolved, as clients may have a number of competing demands that they are juggling and balancing [53].

## Limitations

Our study was contextualized within a country that provides free or low-cost mental health and social services. The outreach and low threshold services were developed in the context of perinatal substance use. We performed a COREQ evaluation for reporting the results [48]. The anonymous feedback was collected by the HTTS organization, and feedback was not available from all clients, which is a restriction of the study. The anonymous client feed-back represented 493 families and 719 parents, yet we do not know the reasons for the 215 clients (30%) out of the 719 persons for not responding. The findings are skewed towards those who completed the program, and who most likely had a positive experience with it and with their recovery.

The staff participated via online surveys, which was feasible as they could respond at any time from different parts of Finland. The narratives were saved online and were easily accessible for the analysis, we can recommend this option of online written narratives. The responses were thorough, and several workers expressed, that it required them quite long to reply in detail. They could write their narratives during their working hours of ETMA.

In the analysis we focused solely on engagement and did not have an intention to find correlations, but to describe building relationships and engagement, and use the results as a 1st step in theory building. Using one coder in this predefined and focused context produced descriptive themes, which were discussed and verified in the research group, where three persons worked within HTTS, and one especially within ETMA. The themes are presented in detail to explicate the complexity of the encounters, and we presented quotations to illustrate the themes.

## Conclusion

The outreach work was characterized by flexibility and the creation of a connection. The low-threshold work aimed at establishing trust, building relationships and engagement, and can use existing interventions such as motivational interviewing. Building a connection, relationships and engagement occurred via physical presence and psychological availability (Fig. 1). The four cornerstones we identified were professional attitudes enhancing engagement: acceptance, attitude, strictness and flexibility. Availability occurs in a specific space, where the everyday life changes require constant and even challenging negotiations and much of the work within outreach and low threshold is carried out by doing things together. Building relationships during pregnancy were characterized by connecting within everyday life situations and supporting the development of an attachment relationship between the baby and the parents. To promote recovery, a comprehensive approach in which substance-related issues and mental health conditions are interconnected is favored. Soon-to-be mothers might be more effectively encouraged to test and visit early preventive intervention programs by well-baby clinics. To help women with SUD during pregnancy, the special perspective of the HTTS and ETMA programs might be of interest to other service providers and function as an asset for developing general services and connections with CPSs. Building connections in a gender-sensitive manner and within trauma-informed approaches could add to the understanding of SUD outreach and low threshold work during pregnancy. The elements described in this study need further theoretical development, research and critical assessment. Engaging early on during pregnancy might enhance success during future rehabilitation.

## Data Availability

The data cannot be openly available in data repositories because the data contain sensitive issues. The data to support the findings are available upon request from the corresponding author.

## Abbreviations

SUD: Substance Use Disorder
HTTS: Holding Tight Treatment System
ETMA: Low threshold outreach program (Etsivän ja matalan työn palvelut)
